# Supporting patients with long-term problems after Lyme disease

**DOI:** 10.3399/bjgpopen20X101102

**Published:** 2020-06-17

**Authors:** Victoria Cairns

**Affiliations:** 1 Consultant Statistician, Oxford, UK

**Keywords:** Post treatment Lyme disease syndrome, Post-Lyme Disease Syndrome, Lyme disease, General practice, Primary health care

There has been controversy about Lyme disease (LD), first about whether people really can have persistent problems after Lyme disease, and then about whether more antibiotic treatment is beneficial if they do still have symptoms. This overview compiles into one summary information about what the studies have found about the cause of the symptoms and the possible treatments, so that GPs are better able to advise patients who have the long-term problems.

Some patients have persistent symptoms after LD, despite having had the recommended antibiotic treatment. A small proportion of LD patients have a relevant delay in treatment, which may increase their risk for the development of problems that can last a long time, perhaps a few years. These delays can sometimes result from the difficulty of diagnosis, particularly as many LD cases do not have the erythema migrans rash.^1^ Catching LD while holidaying abroad may make a delay more likely because it may be some time before the patient returns home and sees a doctor. The Royal College of General Practitioners has prepared an LD toolkit^2^ and mentions post-treatment Lyme disease syndrome (PTLDS) as one term used to describe these long-term problems. PTLDS can significantly impact quality of life^3^ and recovery may be extremely slow, with no apparent improvement from one month to the next, although very subtle improvement may be seen from one year to the next.

PTLDS results in extreme fatigue, cognitive disorders, and musculoskeletal pain. The fatigue has been described as profound and debilitating. The musculoskeletal pain is often roving, asymmetrical pain in the limbs, and is unlike fibromyalgia.^4^ Memory problems, particularly verbal memory, are also observed, as well as poor concentration, including auditory attention. A study by Fallon *et al* has shown that patients with PTLDS demonstrated significant flow reductions in white matter areas in the brain, and that the flow reductions were significantly associated with deficits in memory and visuospatial organisation.^5^ There is some symptom overlap with chronic fatigue syndrome (CFS), but the symptoms of PTLDS, including fatigue, can be clinically significant and long-lasting,^3^ whereas a key symptom of CFS is reported to be post-exertional malaise lasting more than 24 hours,^6^ and there are other differences. A review by Uhde *et al* pointed out that there is evidence of inflammation from the marker C-reactive protein seen with PTLDS, but not with CFS.^7^

Sometimes, after eradication of the actively dividing LD spirochete, there may be debris remaining which can explain some of the long-term symptoms. A study by Jutras *et al* identified a chemically typical peptidoglycan as a likely contributor to inflammatory responses in Lyme arthritis. Persistence of this antigen in the joint may contribute to synovitis after antibiotics eradicate the pathogen.^8^ A study by Parthasarathy *et al* showed that non-viable LD bacteria (heat-killed or sonicated bacteria) induced inflammation and apoptosis of human oligodendrocytes, and they concluded that residues left after bacterial demise may continue to be pathogenic to the central nervous system.^9^ The expression chronic LD may be misunderstood, and some people may mistakenly think they have ongoing spirochetal infection requiring extensive antibiotic treatment, while their symptoms may be from the debris of morphological forms of LD bacteria that are resistant to antibacterial therapy, and those patients may be endangering their health by taking prolonged courses of antibiotics that can lead to other infections.^10^

In a study of autopsy tissues from a patient who had died after many years of antibiotic treatment for LD, Sapi *et al* found evidence indicating that the LD bacteria *Borrelia burgdorferi* can persist in the human body in the antibiotic-resistant bioﬁlm form, even after long-term antibiotic treatment. They found evidence in many parts of her body and the presence of inﬁltrating lymphocytes in the vicinity of *Borrelia burgdorferi* bioﬁlms, which suggests that the organism in bioﬁlm form might trigger chronic inﬂammation.^11^ They wrote that *Borrelia* antigens and DNA are associated with bioﬁlms, and that one hypothesis for their persistence is that diﬀerent morphological forms of *Borrelia* may protect the bacteria from antibacterial therapy.

Since antibiotics do not appear to be effective against these forms of *Borrelia*, other kinds of treatment are needed. Socarras *et al* did an in vitro study on the antimicrobial activity of bee venom and its component melittin against *Borrelia burgdorferi* persisters, and their findings showed that bee venom melittin had significant effects on all the tested forms of *Borrelia burgdorferi*.^12^ Feng *et al* have tested various botanical and natural products, and found several to be highly active in vitro against what they called stationary phase *Borrelia burgdorferi*. The authors concluded that future studies should be directed at identifying the active constituents of each botanical, evaluating synergistic combinations, and conﬁrming their safety and eﬃcacy.^13^

Continuing with antibiotic treatment may affect the immune system in a variety of other ways. Bernardino *et al* did a study on monkey brain cells stimulated with LD bacteria and showed that doxycycline moderated the inflammatory response.^14^ They concluded that, in people with PTLDS who experience a beneficial effect from retreatment with doxycycline, this effect may be due to the anti-inflammatory properties of the antibiotic.

A review by Balducci *et al* highlights that the antibiotic tetracyclines, besides having antimicrobial activity, also have pleiotropic action against amyloidosis, neuroinflammation, and oxidative stress.^15^

A study by Fallon *et al* found that some of the PTLDS patients in their trial experienced moderate short-term cognitive improvement from ceftriaxone treatment.^16^

Tai *et al* have reviewed studies of ceftriaxone which demonstrated that ceftriaxone can have the eﬀect of increasing glutamate transporter-1 expression and glutamate reuptake, thereby suppressing excitotoxicity, and that it also enhances neurogenesis and recovery of neuronal density.^17^ Since ceftriaxone has been shown to have these effects, it is not surprising that some patients with PTLDS may have had some slight improvement in their levels of fatigue and their cognition deficits after a repeated course of ceftriaxone.

The challenge can be in determining whether previous antibiotic treatment has effectively killed the actively dividing LD spirochete, as direct tests are not always accurate or available to clinicians. The oral antibiotic doxycycline is usually effective for early LD, leaving the patient with no further problems, but oral antibiotics may not be effective if the infection has reached the later stages of the disease, when intravenous ceftriaxone may be recommended. Some specialists have said that if the patient is getting better, however slowly, then this is a sign that no further antibiotic treatment is needed.

In many patients with persistent symptoms, the symptoms fade away very slowly over time, and so it appears that there is some gradual recovery taking place, which may be neurological recovery, eventual breakdown of the debris, or a slow cessation of the chronic inflammation.

There are insufficient studies from which to draw definitive conclusions regarding the efficacy of repeated antibiotic therapy. For many patients, further treatment does not appear to provide much benefit, but it may be appropriate for those patients where it is thought that the actively dividing LD spirochete might not have been eradicated; that is a difficult judgement, however.

Instead of receiving repeated antibiotics, which bring some risk and appear to provide little benefit to patients with PTLDS, there are some food supplements that may help to some extent with recovery.

Natural products are sometimes good as therapeutic agents, particularly given their minimal side effects. Manuka honey has been found to have various biological properties, including antioxidant and antimicrobial capacities.^18^ It can react with macromolecules such as DNA, RNA and proteins, without any damage to host cells. Omega-3 fatty acids that are found in natural fish oil have been shown to help with minor cognitive impairment,^19^ and vitamin B12 has been shown to be beneficial in nerve regeneration.^20^ The spice turmeric, also known as curcumin, may help reduce joint inflammation,^21^ and Sirtuin 3 has been identified as a key regulator of mitochondrial fission, and it may help reduce neuroinflammation.^22^ The Sirtuin enzymes are activated by foods like green tea, dark chocolate, and blueberries.

The regrowth of neural pathways is, however, extremely slow, and recovery will inevitably take a long time, with or without any such supplements. The potential harm from inappropriate doses of alternative therapies also needs to be recognised. Nevertheless, patients may feel encouraged if they hear of anything that they can do which may help their recovery, even if only a little. It has not been shown whether any of the above products could help with the long-term recovery from PTLDS, but the main message for those suffering from persistent problems after having had the recommended antibiotic treatment is that PTLDS is recognised, and that many people do recover with time.
